# Effects of Polyphenols from Grape Seeds on Renal Lithiasis

**DOI:** 10.1155/2015/813737

**Published:** 2015-03-25

**Authors:** Felix Grases, Rafel M. Prieto, Rafel A. Fernandez-Cabot, Antonia Costa-Bauzá, Fernando Tur, Jose Juan Torres

**Affiliations:** ^1^Laboratory of Renal Lithiasis Research, University Institute of Health Sciences Research (IUNICS), and University of Balearic Islands, Ctra. de Valldemossa Km 7.5, 07122 Palma de Mallorca, Spain; ^2^CIBEROBN (CB06/03), Instituto de Salud Carlos III, C/ Monforte de Lemos 3-5, 28029 Madrid, Spain; ^3^Hospital Comarcal de Inca, IB-Salut, C/ Llubí S/N, 07300 Inca, Balearic Islands, Spain

## Abstract

Nephrolithiasis is a complex disease that results from a combination of factors related to both urine composition and kidney morphoanatomy. Development of calcium oxalate monohydrate papillary calculi is linked to initial subepithelial calcification of renal papilla. Progressive tissue calcification depends on preexisting injury and involves reactive oxygen species. Many plant extracts that protect against oxidative stress manifest antilithiasic activity. Our study focused on determining the effects of polyphenols on a lithiasis rat model. Rats were pretreated with polyphenols and grape seed extracts, followed by posterior induction of hyperoxalosis via treatment with ethylene glycol plus NH_4_Cl. The concentrations of calcium and other elements in kidney were determined, along with histological examination of kidney and 24 h urine analysis. Significant differences were observed in the renal calcium content between the control plus ethylene glycol-treated group and the epicatechin plus ethylene glycol-treated, red grape seed extract plus ethylene glycol-treated, and white grape seed extract plus ethylene glycol-treated groups, with reductions of about 50%. The antioxidant activity of polyphenols extracted from red and white grape seeds may be critical in the prevention of calcium oxalate monohydrate papillary calculus formation, particularly if calculi are induced by lesions caused by cytotoxic compounds with oxidative capacity.

## 1. Introduction

Renal lithiasis is a disease with increasing prevalence and significant global variations (4–15%) [1]. Nephrolithiasis is a complex disease that results from a combination of various factors related to both urine composition and kidney morphoanatomy. Calcium oxalate is the predominant component of renal stones (70%). Two types of calcium oxalate stones have been distinguished, composed of either calcium oxalate dihydrate (COD) or calcium oxalate monohydrate (COM). COD crystals are thermodynamically unstable and develop only under kinetically favorable conditions, such as a high degree of supersaturation (high calcium concentration >170 mg/L and/or hyperoxaluria), low concentrations of crystallization inhibitors (phytate, citrate), and urodynamically appropriate conditions (e.g., urinary stagnation). Due to thermodynamic instability, COD crystals slowly transform to stable COM crystals mainly in contact with urine [2]. COM calculi, in which crystals are directly formed from urine, are classified into two types [3]. COM papillary calculi (13% of urinary stones) that develop attached to papillary tissue and COM calculi in renal cavities (16% of renal stones). The development of COM papillary calculi is linked to initial subepithelial calcification of renal papilla. Disruption of the epithelial layer by hydroxyapatite (HAP) deposits becomes the nidus of a COM papillary calculus [4–8]. Preexisting injury acts as an inducer of tissue calcification development and continuation of this process depends on the activity of modulators (immune system) and/or deficiency in crystallization inhibitors [9–15]. The tissue calcification process is similar to extracellular matrix mineralization at other tissue localizations (such as vascular tissue) and involves reactive oxygen species and oxidative stress [16–18]. In fact, many plant extracts that protect against the development of oxidative stress manifest clear antilithiasic activity [16–19]. The main aim of the current study was to determine the effects of polyphenols on development of COM papillary renal calculi in a rat model.

## 2. Materials and Methods

### 2.1. Animals and Treatments

Sixty-four male Wistar rats, each weighing approximately 300 g, were acclimatized for 7 days in cages, prior to the experiment. Animal experiments were performed in accordance with general guidelines approved by our institutional ethics committee and EU regulations (86/609/CEE and 2003/65/CE). Rats had* ad libitum* access to standard food and mineral water under a controlled 12 h light/dark cycle at 22 ± 2°C.

Animals were divided into eight groups (eight animals per group) as follows: control without pretreatment (CTR), control plus ethylene glycol (CTR + EG), epicatechin pretreated (EPI), epicatechin plus ethylene glycol-treated (EPI-EG), red grape seed extract pretreated (RGS), red grape seed extract plus ethylene glycol-treated (RGS-EG), white grape seed extract pretreated (WGS), and white grape seed extract plus ethylene glycol-treated (WGS-EG) groups. (−)-Epicatechin >90% (HPLC) was supplied by Sigma-Aldrich (Madrid, Spain), red grape seed extract (exGrape seed) by La Gardonnenque-Groupe Grap'Sud (Cruviers-Lascours, France), and white grape seed extract (Oxvit) by Output Trade S.L. (Vilafranca del Penedès, Spain).

The red grape seed extract is characterized by polyphenols >55% (equivalent of gallic acid), proanthocyanidin >30% (vanillin assay), and oligomer proanthocyanidin content (OPC) or di-, tri-, and tetramer proanthocyanidins >30% (by HPLC assay). The white grape seed extract is characterized by polyphenols >45% (equivalent of gallic acid), proanthocyanidins >35% (by HPLC assay), and OPC >6% (by HPLC assay). The characterization of the extracts is from suppliers specifications.

During a 16-day period, CTR and CTR-EG groups were supplied with drinking water, EPI and EPI-EG groups with drinking water supplemented with 200 mg/L of epicatechin, RGS and RGS-EG groups with drinking water supplemented with 200 mg/L of red grape seed extract, and WGS and WGS-EG groups with drinking water supplemented with 200 mg/L of white grape seed extract. After 16 days, drinking water for rats in the CTR-EG, EPI-EG, RGS-EG, and WGS-EG groups (containing the corresponding pretreatment in each case) was supplemented for further eight days with 0.8% v/v ethylene glycol (EG) plus 1% w/v NH_4_Cl. The remaining groups continued with the corresponding pretreatment for this time-period. Polyphenols were administered in drinking water because their bioavailability can improve compared to administration by a single daily dose [20, 21].

### 2.2. Monitoring and Sampling

Animal weights and drinking water volume were monitored throughout the study every two days. At the end of the experiment, 24 h urine samples were collected from each group of rats in metabolic cages with overnight fasting. On the last day, animals were sacrificed via CO_2_ inhalation, and their kidneys removed for histological and chemical analyses.

### 2.3. Determination of Calcium and Other Elements in Kidney

The left kidneys were lyophilized, weighed, and digested in a dry bath at 180°C using a 1 : 1 HNO_3_ : HClO_4_ mixture until the solution was clear. For chemical analysis, digested samples were diluted with distilled water until 10 mL of solution was obtained, and the concentrations of calcium, phosphorus, and magnesium determined using inductively coupled plasma atomic emission spectrometry (PerkinElmer SL, Optima 5300 DV Spectrometer) and the appropriate calibration curve. The concentrations of calcium and other elements were determined as mg/g kidney dry weight.

### 2.4. Histological Examination

Representative tissue samples from each group were examined with light microscopy. The right kidneys were placed in 4% buffered formaldehyde at pH 7 (supplied by Panreac Quimica S.A., Barcelona, Spain), fixed for 24 h at room temperature, embedded in paraffin wax, sectioned (2–4 *μ*m), and stained with hematoxylin-eosin (H-E). Crystals were observed and localized using polarized light. Histological analysis and crystal localization were performed by an experienced pathologist. The crystallization score was obtained by averaging all of the scores from all sections for all H-E stains. Scores from 2 to 6 sections per stain per sample were determined and averaged. The scores were graded from 0 to 3+: 0 = no crystallization; 1+ = mild crystallization; 2+ = moderate crystallization; and 3+ = severe crystallization.

### 2.5. Urine Analysis

Overall, 24 h urine samples were analyzed for calcium, magnesium, and phosphorus content using inductively coupled plasma atomic emission spectrometry; creatinine excretion was determined by the Jaffe method, and the oxalate content determined using an oxalic acid kit (supplied by Spinreact SAU, St. Esteve d'en Bas, Girona, Spain). The pH of each sample was measured using a glass electrode (pH meter, Crison S.L., Barcelona, Spain).

### 2.6. Statistical Analysis

Results are presented as mean values ± SEM of eight animals per group. Statistical differences between groups were analyzed with Student's *t*-test, and *P* values less than 0.05 considered statistically significant. Conventional Windows software was used for statistical computations.

## 3. Results

### 3.1. Body Weight and Water Intake

No significant differences were found in body weight and drinking water volume consumption between pretreated groups during the first 16 days of polyphenols pretreatment. EG/NH_4_Cl-treated groups showed lower body weight and drinking water volume due to the toxic effects of treatment.

### 3.2. Calcium and Other Elements in Kidney

The calcium, magnesium, and phosphorus concentrations determined from left kidney tissues are shown in Table 1. Calcium concentrations were significantly higher in the CTR-EG-, EPI-EG-, RGS-EG-, and WGS-EG-treated groups, compared with their respective control groups (CTR, EPI, RGS, and WGS). Moreover, significant differences were observed between CTR-EG and the pretreated (EPI-EG, RGS-EG, and WGS-EG) groups, with reductions of 42%, 56%, and 50%, respectively, in terms of kidney calcium content. No significant differences were evident in the magnesium and phosphorus concentrations in kidney.

### 3.3. Histology of Renal Papillary Tissue

Three representative tissue samples from each group were examined with light microscopy. The kidney sections of rats not administered with EG/NH_4_Cl showed no polarized crystal deposits, while all rats treated with EG/NH_4_Cl exhibited intratubular crystal deposits compatible with calcium oxalate detected with polarized light. We observed the greatest quantity of crystals in the CTR-EG group, similar amounts in the EPI-EG group, and lower crystallization in the WGS-EG group. The RGS-EG group contained small crystal deposits that were not detected with polarized light compatible with hydroxyapatite crystals. These results are summarized in Table 2.

### 3.4. Urine Analysis

The concentrations of the main urinary biochemical parameters of rats in each group are shown in Table 3. Overall, EG/NH_4_Cl treatment resulted in significant diminution of diuresis for each group, increase in oxalate excretion and oxalate urinary concentration, decrease in calcium urinary concentration, and increase in magnesium and phosphorus concentrations relative to the respective pretreatment groups. A significant increase in oxalate excretion and concentration in urine were evident in the RGS-EG and WGS-EG groups, compared to the CTR-EG and EPI-EG groups. A significant diminution of creatinine excretion for CTR-EG and EPI-EG group, relative to the respective pretreatment groups, was observed. Finally, a significant increase in creatinine excretion in urine was evident in the EPI-EG, RGS-EG, and WGS-EG groups, compared to the CTR-EG group.

## 4. Discussion

EG plus NH_4_Cl-induced renal lithiasis model has been extensively used in numerous studies of calcium oxalate nephrolithiasis [22]. This model clearly presents a loss of renal function that can be related to a decrease in urinary creatinine excretion and diuresis [23]. The lithogenic effect of EG may be attributable to oxidative injury caused by the high level of oxalate generated as a result of EG ingestion and thus constitutes a good model for evaluation of COM papillary stone formation.

Polyphenols are antioxidants present in the diet. The main dietary sources are fruits and fruit juices. Tea, red wine, vegetables, legumes, cereals, and chocolate also contribute to the total polyphenol intake, which could reach 1 g/day in humans [24]. Studies focusing on the antioxidant properties of polyphenols were initiated relatively recently (1990s). Evidence obtained to date supports a contributory role of polyphenols in the prevention of cardiovascular disease, some cancer types, and osteoporosis [25].

In a previous paper, which investigated the protective effects of several antioxidant compounds, as catechin, against the oxidative stress associated with renal failure induced by EG plus NH_4_Cl model, it was found that catechin pretreatment improved oxidative status by enhancing antioxidant defenses—superoxide dismutase and PON1 activities—and reducing oxidative damage. The oxidative damage protection exerted by catechin involved an induction of SOD activity [26].

In the present study, we investigated the effects of the well-known polyphenol, epicatechin, on EG plus NH_4_Cl-induced renal lithiasis in rats, in comparison with two polyphenolic extracts obtained from red and white grape seeds. Upon pretreatment with both polyphenolic extracts, calcium deposition in EG-treated rat kidneys was significantly reduced to the same levels of those observed in rats treated with epicatechin. The results show an improvement of the renal function in some treated groups with an increase of diuresis and creatinine excretion due to polyphenols pretreatment. Our results clearly validate the ability of polyphenols to protect against papillary calcification of kidney tissue, consequently preventing the development of papillary calculi, consistent with earlier findings on the positive effects of polyphenols extracted from green tea on oxidative damage in rat kidney [27]. The green tea polyphenol, epigallocatechin-3-gallate, has been shown to improve immune-mediated glomerulonephritis in mice [28]. Resveratrol reduces renal ischemia reperfusion injury through a nitric oxide-dependent mechanism in male Wistar rats [29]. Other studies using resveratrol revealed improvement of microcirculation and protection of tubular epithelium in a mouse model of sepsis-induced acute kidney injury [30]. Another investigation evaluated the antioxidant defense system of rat kidney following chronic exposure to red wine rich in polyphenols [31]. Plasma antioxidant capacity was evaluated based on the ferric reducing ability of plasma (FRAP), and reduced glutathione (GSH) and glutathione disulfide (GSSG) levels were determined. The increase in FRAP and GSH/GSSG ratios was attributed to the polyphenols in red wine [31].

The collective findings to date support a protective effect of polyphenols on kidney tissue. Thus, considering that injury of papillary tissue constitutes the first step in the development of COM papillary calculi, the antioxidant action of polyphenols may contribute significantly to their antilithiasic action. In fact oxidative stress is clearly associated with injury to papillary tissue, resulting in intrapapillary calcifications [32]. Oxidative stress is a result of excessive production of free radicals and the failure of antioxidant defense mechanisms that protect cells by removing free radicals. These antioxidants (that can be supplied through the diet or by a food supplement) could play an important role in avoiding the intrapapillary calcifications, induced by oxidative stress, which are the necessary initial stage of the COM papillary calculi formation, one of the most prevalent renal calculi [3]. Studies of the mechanism of COM papillary formation began with the finding that a subepithelial calcification of renal papilla, resulting from the disruption of the papillary epithelial layer by a hydroxyapatite (HAP) plaque, becomes the nidus of a COM papillary calculus [4–8, 33]. The direct contact between HAP and urine (always supersaturated in calcium oxalate) initiates the formation of columnar COM crystals through heterogeneous nucleation, generating the main calculus body. In patients susceptible to papillary calculi, the plaque is initiated in thin-loop basement membranes, in basement membranes of collecting tubules, and in* vasa* recta [4–6, 33] due to cell damage caused by reactive oxygen species and oxidative stress [34–36]. Crystals in Randall plaques have been associated with collagen and membrane bound vesicles [37], suggesting that Randall plaque formation is similar to extracellular matrix mineralization at other body locations and involves reactive oxygen species and oxidative stress [34–36]. As a conclusion, the antioxidant activity of polyphenols extracted from red and white grape seeds can play an important role in prevention of COM papillary calculi, particularly avoiding papillary tissue injury caused by cytotoxic substances with oxidative capacity. As is demonstrated, this antioxidant activity prevents oxidative membrane and DNA damage through metal chelation and reactive oxygen species scavenging [38].

## Figures and Tables

**Table 1:** Kidney content of calcium, magnesium, and phosphorus.

Pretreatment	EG treatment	Calcium	Magnesium	Phosphorus
		(mg/g)	(mg/g)	(mg/g)
CTR		0.280 ± 0.005	0.302 ± 0.006	10.6 ± 0.2
EPI		0.284 ± 0.030	0.296 ± 0.014	10.2 ± 0.4
RGS		0.255 ± 0.008^a^	0.288 ± 0.004	10.1 ± 0.1
WGS		0.261 ± 0.012	0.295 ± 0.004	10.3 ± 0.2
CTR	EG	3.951 ± 0.934^*^	0.297 ± 0.011	10.4 ± 0.1
EPI	EG	2.274 ± 0.447^∗,a^	0.302 ± 0.010	9.9 ± 0.4
RGS	EG	1.734 ± 0.564^∗,a^	0.311 ± 0.005^*^	10.5 ± 0.1
WGS	EG	1.979 ± 0.196^∗,a^	0.293 ± 0.010	9.9 ± 0.3

Values represent mean ± SEM of eight animals per group. (CTR): control with no pretreatment, (EPI): epicatechin pretreatment, (RGS): red grape seed extract pretreatment, (WGS): white grape seed extract pretreatment, and (EG): ethylene glycol/NH_4_Cl treatment.

^*^Values significantly different to the respective pretreated groups, *P* < 0.05; ^a^values significantly different to CTR, *P* < 0.05.

**Table 2:** Histology data from renal papillary tissue (*n* = 3).

Pretreatment	EG treatment	Crystal deposits
CTR		0
EPI		0
RGS		0
WGS		0
CTR	EG	+++
EPI	EG	+++
RGS	EG	+
WGS	EG	++

(CTR): control with no pretreatment, (EPI): epicatechin pretreatment, (RGS): red grape seed extract pretreatment, (WGS): white grape seed extract pretreatment, and (EG): ethylene glycol/NH_4_Cl treatment.

0 = no crystallization; + = mild crystallization; ++ = moderate crystallization; and +++ = severe crystallization.

**Table 3:** Urinary biochemical data at the end of experiment, following 8 days of ethylene glycol/NH_4_Cl treatment.

Pretreatment	EG treatment	Diuresis	Creatinine	pH	Oxalate	Calcium	Magnesium	Phosphorous
		(mL/24 h)	(mg/24 h)	(units)	(mM)	(mM)	(mM)	(mM)
CTR		30.7 ± 1.7	11.6 ± 0.3	7.2 ± 0.1	0.21 ± 0.02	0.56 ± 0.05	0.45 ± 0.05	14.20 ± 1.06
EPI		24.9 ± 2.1	9.3 ± 0.3	7.2 ± 0.0	0.24 ± 0.02	0.50 ± 0.05	0.44 ± 0.06	15.67 ± 1.31
RGS		25.4 ± 0.6	9.1 ± 0.3	7.3 ± 0.1	0.21 ± 0.01	0.51 ± 0.02	0.47 ± 0.07	15.78 ± 0.65
WGS		27.8 ± 1.5	10.6 ± 0.3	7.3 ± 0.1	0.23 ± 0.02	0.53 ± 0.07	0.46 ± 0.06	13.17 ± 0.70^c^
CTR	EG	9.0 ± 3.5^*^	4.0 ± 0.1^*^	7.1 ± 0.5	1.32 ± 0.13^*^	0.29 ± 0.06^*^	1.70 ± 0.43^*^	50.51 ± 11.11^*^
EPI	EG	7.8 ± 1.6^*^	6.8 ± 0.3^a,∗^	6.3 ± 0.0^*^	1.59 ± 0.08^*^	0.10 ± 0.03^∗,a^	1.51 ± 0.15^*^	23.07 ± 2.77^∗,a^
RGS	EG	12.3 ± 1.6^∗,b^	8.2 ± 0.4^a^	7.6 ± 0.5^b^	2.69 ± 0.63^∗,a^	0.08 ± 0.03^∗,a^	1.09 ± 0.15^∗,b^	34.65 ± 3.15^∗,b^
WGS	EG	16.0 ± 1.3^∗,b^	10.1 ± 0.2^a^	6.2 ± 0.1^∗,a,c^	2.17 ± 0.12^∗,a,b^	0.04 ± 0.01^∗,a,b^	1.47 ± 0.28^*^	33.87 ± 2.83^∗,b^

(CTR): control not pretreated, (EPI): epicatechin pretreated, (RGS): red grape seed extract pretreated, (WSE): white grape seed extract pretreated, and (EG): ethylene glycol/NH_4_Cl treatment.

Values represent mean ± SEM; ^*^values significantly different to the respective pretreated group, *P* ≤ 0.05; ^a^values significantly different to CTR, *P* ≤ 0.05; ^b^values significantly different to EPI, *P* ≤ 0.05; ^c^values significantly different to RGS, *P* ≤ 0.05.
